# Could Wearable and Mobile Technology Improve the Management of Essential Tremor?

**DOI:** 10.3389/fneur.2018.00257

**Published:** 2018-04-19

**Authors:** Jean-Francois Daneault

**Affiliations:** Motor Behavior Laboratory, Department of Rehabilitation and Movement Sciences, School of Health Professions, Rutgers, The State University of New Jersey, Newark, NJ, United States

**Keywords:** essential tremor, smartphone, smartwatch, mobile, wearable, technology, movement disorder

## Abstract

Essential tremor (ET) is the most common movement disorder. Individuals exhibit postural and kinetic tremor that worsens over time and patients may also exhibit other motor and non-motor symptoms. While millions of people are affected by this disorder worldwide, several barriers impede an optimal clinical management of symptoms. In this paper, we discuss the impact of ET on patients and review major issues to the optimal management of ET; from the side-effects and limited efficacy of current medical treatments to the limited number of people who seek treatment for their tremor. Then, we propose seven different areas within which mobile and wearable technology may improve the clinical management of ET and review the current state of research in these areas.

## Introduction

Essential tremor (ET) is a movement disorder where individuals exhibit bilateral, persistent postural, or kinetic upper-limb tremor (1–4) that can also be observed during rest in some cases (4–9). Tremor amplitude varies greatly between patients and also within one patient from day to day, and within a given day (10, 11). Interestingly, tremor amplitude tends to increase with advancing age (12, 13). To date, diagnosis of ET is based on clinical examination and neurological history. Very recently, the International Parkinson and Movement Disorders Society Task Force on Tremor has proposed that individuals exhibiting symptoms other than tremor should be labeled as having ET plus syndrome (14). These accompanying neurological signs may include mild to moderate gait ataxia (3, 15–23), cognitive impairment (24, 25) as well as personality profile (26–28), and mood disturbances (29–31). Thus, ET is no longer considered a benign form of tremor but rather a complex disorder.

ET is the most prevalent movement disorder among adults (32). Its prevalence is markedly higher than that of Parkinson’s disease (PD) (33–35) and many “common” neurological diseases such as epilepsy, stroke, and multiple sclerosis (36). The overall prevalence of ET is estimated at 0.9% (37); suggesting that there are about 70 million individuals with ET worldwide. Prevalence increases markedly with age; such that a rate of 4.6% in individuals 65 years and older, and a rate of up to 20% in the oldest old was estimated (37). Therefore, due to the aging population and extended life expectancy, ET is becoming a larger cause of functional disability.

### What Is the Impact of ET on Quality of Life?

Tremor negatively impacts activities of daily living (ADLs) (38); and can lead to embarrassment and stigmatization affecting emotional well-being (39, 40). Mild functional impairment due to tremor has been reported in 60–73% of individuals with ET (31, 41) while moderate to severe impairment in everyday life has been reported by 26% of patients (31). Of note, 5.3% of individuals with ET reported that they frequently needed help in ADLs because of their tremor. Therefore, millions of individuals experience daily functional impairments due to ET.

Non-motor symptoms, such as depression and anxiety, are also often observed in individuals with ET; impairing their quality of life (41–43). Higher levels of anxiety are observed among individuals with ET than in healthy individuals (28), and major depressive disorder was diagnosed in 5.4% of individuals with ET whereas it was only observed in 2.7% of healthy individuals (41). In patients with ET, depression has been largely attributed to the impact of tremor on ADLs, employment and hobbies as well as the embarrassment caused due to tremor during social interactions. ET can often force individuals to retire or change profession, and become reluctant to leave their homes (31). Taken together, this indicates that tremor severity and non-motor symptoms of ET significantly impair quality of life.

### What Are the Current Treatments for ET?

Medication is the primary intervention utilized to minimize tremor severity in individuals with ET. While several medications can be prescribed (44), propranolol and primidone are the most frequently used (45). However, the effectiveness of medication for management of tremor in ET is quite variable, likely due to several factors including the multiple etiologies of ET (14).

One option for the management of tremor that fails to respond to traditional drug therapy is the injection of botulinum toxin. This approach has been used successfully with the advantage of providing very targeted relief (46–51). Many clinicians will use it as a first line treatment to improve head tremor (51) while others also use botulinum toxin to manage intractable limb tremor. Injections are a relatively safe treatment option and are usually effective in reducing tremor severity.

Aside from drug therapy and toxin injections, surgical options may be considered in individuals suffering from important functional disability. Lesions (52–54) and deep brain stimulation (DBS) (55–61) of the nucleus ventrointermedius (Vim) and neighboring structures are believed to disrupt ET-related pathological oscillations and thereby reduce tremor severity. Generally, Vim DBS leads to reduction of 80% in tremor severity in about 80% of cases (62, 63).

Another, albeit less conventional, treatment for ET is alcohol. Individuals with ET have a transient diminution of tremor amplitude after drinking alcohol (64–66). Koller and Biary (67) demonstrated that alcohol reduced tremor amplitude by an average of 67%. This response can be observed in a majority of ET patients (68). In addition to reducing tremor amplitude, alcohol was also shown to improve gait ataxia in individuals with ET (69).

Of note, while it is now recognized that patients may not only exhibit tremor but also other motor and non-motor symptoms (ET plus syndrome) (14); and it has been shown that those symptoms negatively impact quality of life, there is no literature examining the specific treatment to address these associated ET symptoms.

### What Are the Main Issues with Current Treatments and Other Factors Limiting Optimal Care?

Only about 50% of patients have lasting benefit from either propranolol or primidone treatment (70–73). Furthermore, tolerance to those drugs has been reported and their side-effects can be dose-limiting (70–73). A more important issue is that there is no significant difference in quality of life in individuals with ET taking medication from those that do not (42); highlighting the variable efficacy of drug treatments.

In addition to the inherent cost of surgical treatments for ET, there are several issues that also limit their use. For instance, although some studies have demonstrated long-lasting effects of Vim DBS (63), others have observed a gradual loss of tremor control in some patients that probably stems from tolerance to stimulation (55, 61, 74–76). There are also side-effects to Vim DBS. While they are usually reduced or eliminated by adjusting the stimulation parameters, they occasionally lead to treatment discontinuation (60). Another issue with DBS for ET is that advanced age is a relative exclusion criterion for the surgery (77). However, it is worth noting that new technological developments in stimulation (e.g. directional and adaptive stimulation) and lesion (e.g. focused ultrasound) technology could soon improve clinical outcomes of surgical interventions.

Self-medication with alcohol also poses problems in ET. For instance, to obtain long-lasting benefits, repeated alcohol intake is necessary (66). Thus, some patients with ET eventually develop alcohol use disorder (78). In fact, an incidence of alcohol use disorder of up to 67% was observed in individuals with ET (78–80). Another issue with alcohol is that it actually minimizes the efficacy of propranolol (81).

The above-mentioned information indicates that the different treatment options for ET come with many potential side-effects. Better understanding the symptoms of ET and the effectiveness of the currently available treatments may improve patients’ quality of life as well as help in the development of novel therapeutic strategies.

Another limiting factor to proper management is that although the prevalence of ET is very high (37), individuals with ET rarely seek medical care for the disorder. Community-based epidemiological studies have observed that only between 1 and 27% of individuals with ET seek medical treatment for tremor (31, 82–84). Of those who did seek medical advice, 15% did so only after significant functional disability had occurred (84). This greatly limits our ability to provide proper care to those individuals.

Finally, the lack of scientific evidence regarding the treatment of symptoms other than tremor in ET plus syndrome is lacking. Therefore, there is a need to further research how to best manage these symptoms to provide optimal outcomes for patients.

## Is it Possible to Improve Evidence-Based Approaches to Manage ET Using Mobile and Wearable Technology?

**Figure 1:**
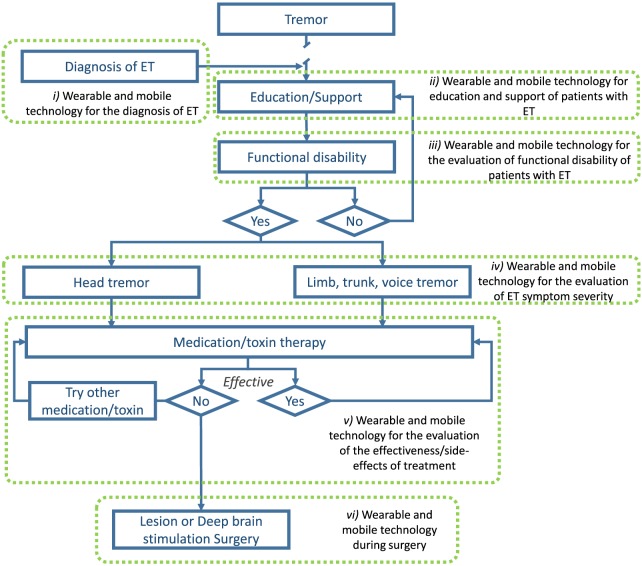
Illustration of areas where wearable and mobile technology could be utilized to improve the management of ET. Adapted from Gironell and Kulisevsky (45) with permission.

Compared to other chronic disorders, there are unfortunately very few studies that have explored the role of mobile and wearable technology in the management of ET. Presented next are seven areas, both within and outside the current treatment algorithm, where mobile and wearable technology may improve patient outcomes (see Figure 1).

### Wearable and Mobile Technology for the Diagnosis of ET

As of today, the most significant barrier to proper management of ET is early and widespread diagnosis. As mentioned above, only a minority of individuals with ET actually seek medical attention and obtain a diagnosis. The ubiquity of technological platforms, such as smartphones and connected devices, may help in the diagnosis of ET. Already, some studies have shown that wearable sensors and smartphones can be utilized to distinguish individuals with ET from healthy individuals and people with PD (85–91). Recent results show an accuracy of 100% in differentiating both disorders (86). With more data and the development of more sophisticated machine learning algorithms, it may be possible to accurately diagnose ET in the general population using wearable and mobile devices.

### Wearable and Mobile Technology for Education and Support of Patients With ET

Although ET is the most prevalent movement disorder, very few people in the general population are familiar with it. As the disease progresses and symptoms exacerbate, social stigma often leads to negative impacts on quality of life. Educating people about the disease process and preparing them to live with this disorder may help them in the long term. Mobile devices can be used to provide educational and support platforms specifically tailored for patients. While, to our knowledge, the effectiveness of such approaches has yet to be addressed for patient education and support in the movement disorder field, there is an opportunity to improve patient quality of life. For instance, studies have demonstrated the effectiveness of using mobile devices to improve health literacy of individuals with other chronic disorders (92–94) which led, in some cases, to improved clinical outcomes (93, 94). Furthermore, recent studies have suggested that peer-to-peer support through social media applications may benefit mental health through interactions with social groups that lead to feelings of belonging and sharing of personal stories/coping strategies (95).

### Wearable and Mobile Technology for the Evaluation of Functional Disability of Patients With ET

The use of wearable technology for the evaluation of functional activities (i.e., ADLs) in patient populations is steadily growing. Recently, groups have employed wearable sensors to assess functional activities in stroke survivors (96–98) and individuals with PD (99, 100). Much work has been done on using wearable sensor data features to identify daily functional activities in unsupervised conditions using simple wearable technology [e.g., Ref. (101, 102)]. Being able to identify “what” patients are doing is the first step in recognizing whether they are impaired or not. The next step is to identify “how” patients are doing ADLs. This is a much more complicated task as it is more subjective, and defining task “success” criteria is problematic at best. Nonetheless, some groups have started considering this by identifying wearable sensor features from daily functional tasks that are associated with the overall frailty level of older individuals (103, 104). While much work remains to be done, mobile and wearable technology could provide important information regarding when ADLs become impaired.

### Wearable and Mobile Technology for the Evaluation of ET Symptom (i.e. Tremor) Severity

As discussed above, mobile and wearable technology is being used to help in the diagnosis of ET. Studies have focused on identifying several tremor characteristics to help differentiate different tremor disorders because ET clinical diagnosis is based on tremor. Several groups have utilized mobile and wearable technology to assess tremor severity in ET without using it for diagnostic purposes [e.g., Ref. (105, 106)] but rather as a tool to monitor the evolution of symptoms over time. This is an area in which technology has an advantage over current clinical approaches because it could provide a better understanding of long-term tremor behavior; over hours, days, weeks, and even months. Clinicians currently use clinical rating scales that can only provide a measure of maximal tremor severity on a very short time window. It is impractical to perform clinical assessments at multiple time points. To get a better understanding of tremor fluctuations over time, clinicians rely heavily on patient recall; which can be misleading (107, 108) and suffer from recollection bias. While mobile technology could enable more frequent tremor assessments, wearable technology could provide continuous tremor assessment (106, 109). This may inform on the pathophysiological mechanisms underlying ET as well as help clinicians in the titration of medications and prescription of more invasive treatment options.

### Wearable and Mobile Technology for the Evaluation of the Effectiveness of Treatment

As clinicians usually rely on a treatment algorithm (45) to achieve the best possible outcome in tremor reduction in ET, it is pivotal to know how effective a given drug or treatment is in minimizing tremor. As mentioned above, the methods currently in use are clinical rating scales and patient reports which both have limitations. Utilizing mobile and wearable technology prior to treatment initiation or before a treatment modification could provide a solid basis to compare data obtained after the change in patient status and identify whether the treatment was effective in significantly reducing tremor severity. This could inform clinicians as to the effectiveness, if any, of the new intervention. The longitudinal data could help in titrating medication dosage or reduce the time patients must wait prior to moving on to another treatment if the current one is not effective by providing large quantities of objective data. By optimizing the treatment of patients, it could minimize the impact ET has on their ADLs and reduce stigma, thus improving their quality of life.

Wearable and mobile technology may also be useful in the identification of treatment side-effects. Side-effects of ET treatment can sometimes include heart problems and balance disorders; both of which could be identified and monitored using wearable technology (110, 111). Recent advances in speech processing (112) also suggest that mobile platforms could be employed to identify and monitor speech characteristics that may be altered by DBS. Wearable and mobile devices therefore have the potential of identifying and monitoring the possible side-effects of ET treatment which could help clinicians optimize treatment outcomes.

### Wearable and Mobile Technology During Surgery

Surgical interventions aimed at alleviating intractable tremor are costly procedures with potentially significant side-effects. It is therefore of the utmost importance to optimize the outcome of such interventions and attempt to minimize their risk. To that end, some groups have started looking into using wearable and mobile technology to support the surgical team in identifying the optimal target location for DBS electrodes (113). While data from wearable devices collected during DBS surgery cannot replace the expertise of an experienced movement disorder specialist, they can provide additional information on objective tremor characteristics to the clinical team about the impact of different stimulation parameters at different locations. This can therefore help surgeons place the lead in a patient-specific optimal location and provide the DBS programming team a good starting point to reduce the time to achieve optimal stimulation parameters. Of note, the same on-line tremor monitoring approach could be utilized for lesion approaches.

### Wearable and Mobile Technology for the Evaluation of ET Symptom Severity Other Than Tremor

As discussed above, patients with ET plus syndrome may exhibit gait ataxia (3, 15–23) as well as cognitive impairment (24, 25), personality profile (26–28), and mood disturbances (29–31). Gait ataxia can be reliably assessed using wearable sensors in individuals with spinocerebellar degeneration (111) and thus, with some work could reasonably also be utilized to assess this issue in patients with ET. On the other hand, much less work has been done to use wearable and mobile technology to assess the severity of non-motor symptoms. Some groups have developed mobile applications to assess cognitive function in different populations (114, 115). While this shows promise, these studies were in the proof-of-principle phase therefore, more work is required to assess whether mobile technology can be used to provide reliable assessments of cognitive function. As for the assessment of personality profile and mood disturbances, there are groups that have monitored physiological signals such as heart rate variability and skin conductance to assess psychological function in different populations (116–118). The very limited evidence concerning the use of mobile and wearable technology for the assessment and management of non-motor symptoms highlights the opportunities in this field. The development of such tools would benefit not only individuals with ET but also several other patient populations.

### Challenges to the Use of Wearable and Mobile Technology for the Management of ET

While the opportunities for the use of wearable and mobile technology in the management of ET are very promising, there are also challenges that need to be addressed before widespread adoption of this technology can be envisioned.

While the storage capacity of commercially available systems is continually expanding and opportunities to store large quantities of data in the cloud become more readily available, our ability to analyze these datasets to provide clinically relevant information to clinicians and patients remains limited at this time (119). Collaborative efforts between engineers, computer scientists, and clinicians are required to identify the relevant information that can be extracted from the data using novel signal processing and machine learning algorithms.

Another important barrier to wider implementation of mobile and wearable technology for home or long-term monitoring is patient compliance. Studies have shown that while patients can utilize wearable sensors for long-term monitoring (120, 121), some patients eventually find those devices uncomfortable and/or burdensome (120). This highlights the fact that, in designing wearable sensors and mobile applications, it may be beneficial to involve the target patient population to optimize those technologies to their needs and expectations.

## Conclusion

ET is already the most common movement disorder and its prevalence will undoubtedly increase over the next years due to the aging population. Because ET is no longer considered a benign disorder and its negative impact on patients’ quality of life has been thoroughly examined, there is now a need to identify new and innovative ways to manage the disease to minimize its burden. Based on the current state of mobile and wearable technology, their ubiquity could be leveraged to improve quality of life of patients if clinicians, engineers, and computer scientists work collaboratively on addressing the current gaps in knowledge and performing larger studies to validate outcome measures.

## Author Contributions

JF-D conceived, designed, and wrote the manuscript.

## Conflict of Interest Statement

The author declares that he owns shares and is a co-founder of Medapplets.
